# Discharge process for patients experiencing homelessness in the emergency department: A thematic qualitative study

**DOI:** 10.1371/journal.pone.0304865

**Published:** 2024-06-07

**Authors:** Elle R. Marcus, Jossie A. Carreras Tartak, Helena Halasz, David Chen, Jarone Lee, Shuhan He

**Affiliations:** 1 Department of Emergency Medicine, Massachusetts General Hospital, Boston, MA, United States of America; 2 Department of Emergency Medicine, Beth Israel Deaconess Medical Center, Boston, MA, United States of America; 3 Department of Emergency Medicine, Newark Beth Israel Medical Center, Newark, NJ, United States of America; 4 Temerty Faculty of Medicine, University of Toronto, Toronto, Ontario, Canada; The University of Alabama, UNITED STATES

## Abstract

People experiencing homelessness are more likely to utilize emergency departments than their non-homeless counterparts. However, obtaining a bed in a homeless shelter for patients can be complex. To better understand the challenges of finding a safe discharge plan for homeless patients in the emergency department, our team conducted interviews with emergency department social workers and homeless shelter case managers in the Boston area. We identified and mapped the stages in the processes performed by both parties, identifying challenges with successful placement into a shelter. Furthermore, we assembled a data dictionary of key factors considered when assessing a patient’s fit for a homeless shelter. By identifying bottlenecks and areas of opportunity, this study serves as a first step in enabling homeless individuals to receive the post-discharge assistance they require.

## Introduction

The COVID-19 pandemic highlighted a critical issue that many urban communities face: providing adequate housing for people experiencing homelessness (PEH). According to the 2022 Boston Homeless Census, on the designated night of the annual count, Boston experienced a total of 1,545 individuals living without permanent shelter [1]. This statistic is a crucial snapshot of the homelessness situation in Boston during 2022, offering a pertinent backdrop for our discussion on homeless patient care at our single center. In Boston, Massachusetts, emergency shelters restricted their bed capacity to per state and city regulations to allow for adequate social distancing. This regulation placed a burden on many essential workers, including the social workers who work in the emergency department (ED) to assist with placing discharged patients in an appropriate outpatient facility.

The population of PEH can be broadly categorized based on their access to shelter, where unsheltered individuals make up a minority population who either opt to not stay in homeless shelters or remain unsheltered due to capacity limits at existing shelters [2]. There remains ongoing debate over the definition of homelessness, ranging from individuals who are precariously housed at high risk of likely homelessness to individuals who are definitively without shelter or housing [3]. For the purposes of this paper, when we use the term PEH, we are referring to people who are unsheltered at the time of their ED visit.

When treating PEH in the ED, disposition is an important consideration; if a patient does not meet admission or respite care criteria, they may be discharged with no stable housing option. For example, a study from an academic center in Philadelphia evaluating ED visits by regular users found that 64% of visits by PEH led to them being discharged to the street [4]. Notably, PEH without stable housing are often released from care without specific housing or shelter support, leading to a positive feedback cycle where released PEH constantly re-enter the emergency department [5]. There exist multi-factorial causes of PEH that contribute to this feedback cycle, including structural factors related to access to medical providers, mental health support, substance use management, and social services that collectively support healthcare infrastructure for PEH [6, 7].

Compounding the problem, PEH often have comorbidities and healthcare needs that can be exacerbated by housing instability. When a PEH is discharged to the street, their nomadic living conditions disrupt the continuity of care required for proper recuperation. They might have inconsistent access to sleep or food or be sleeping in conditions that are cold, wet, and unsanitary [8]. Medication compliance is potentially compromised, as there is no reliable storage for medications, resulting in necessary medications being easily lost [9]. Follow-up care is more difficult to arrange without access to reliable transportation, insurance, or access to phones; [9] and when one is concerned about their next resting place [10]. The cumulative effect of these living conditions is that homeless individuals discharged to the street suffer increased morbidity and mortality [4, 8].

While homeless shelters provide a temporary solution for patients in need of housing after an ED visit, it should be noted that discharging PEH with complex medical needs to a shelter can put a strain on these emergency housing facilities when they are unable to provide the care that these patients need [11]. This underscores the importance of ensuring compatibility between a patient’s medical needs and the facility to which they are being discharged. Furthermore, effective hospital discharge planning can contribute to preventing homelessness by identifying appropriate long-term housing options for PEH [12].

Social workers and case managers are two client-facing health professionals that support the management of social determinants of health for PEH, collectively aiming to improve the patient’s health and well-being. Social workers aim to provide front-line, personalized support to minimize the risks of homelessness in the emergency department. These tasks include connecting PEH with community support services, securing welfare support, and mental encouragement of long-term resilience to overcome homelessness [13]. Shelter case managers aim to provide end-to-end, administrative management of complex health concerns of PEH, including brokering connections to other healthcare providers for multiple cases [14]. Given that these two professions are at the forefront of managing the needs of this complex patient population, we sought to better understand their current process for evaluating the needs of PEH in the ED and upon arrival to a shelter, and to identify barriers in the process of successfully placing PEH in an appropriate shelter or housing situation.

This study has four goals. The first aim is to understand the intake inclusion and exclusion criteria for Boston homeless shelters in order to identify current barriers to shelter placement. The second aim is to describe the discharge process from the perspective of the social worker tasked with facilitating the discharge of patients experiencing homelessness from the ED. The third goal is to describe the emergency shelter intake process from the perspective of shelter case managers as they triage potential clients. The final goal is to highlight actionable roadblocks in the discharge process that would allow us to work towards a more automated and efficient solution for discharging patients experiencing homelessness post-ED visit.

## Methods

**Fig 1:**
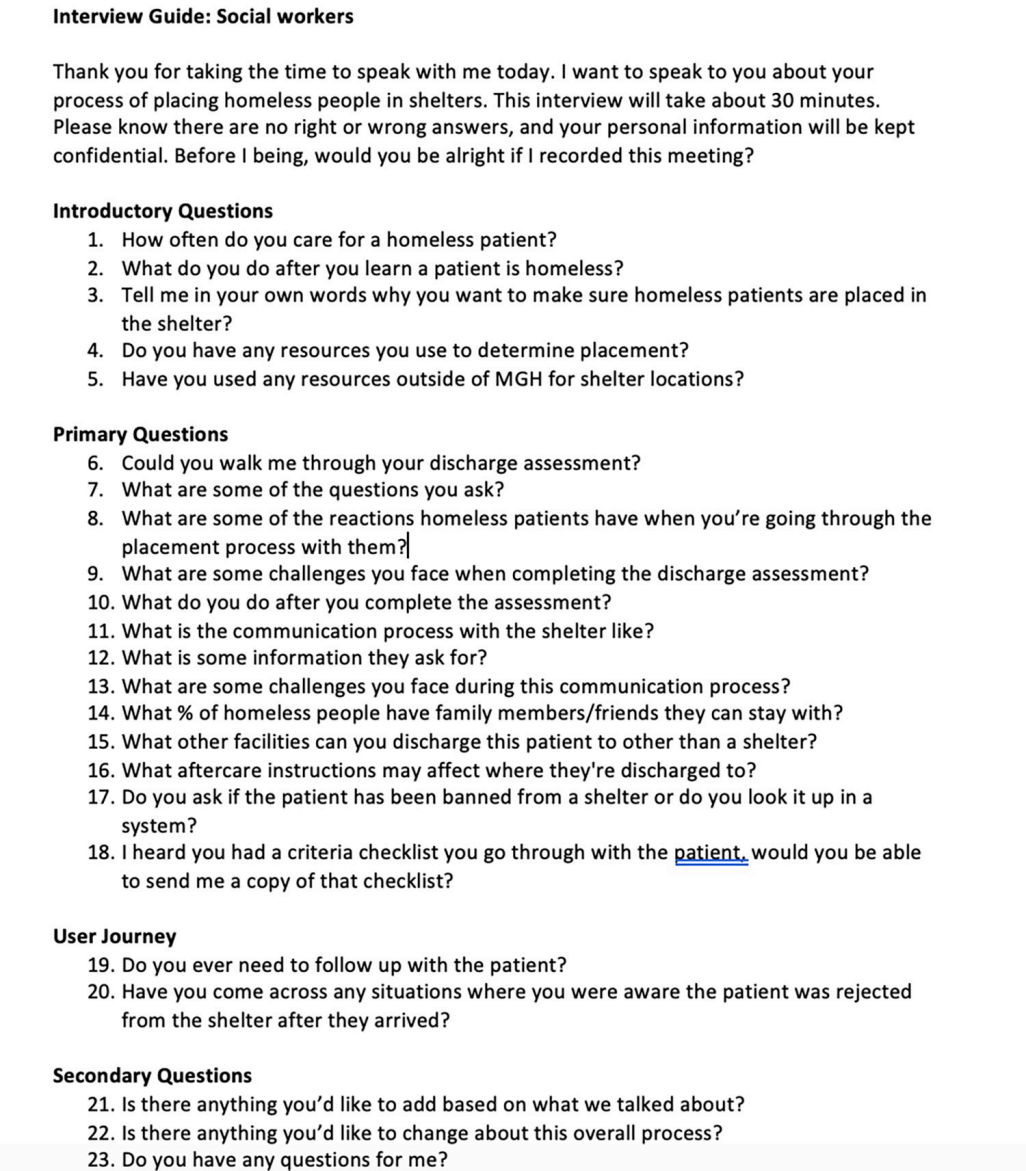
Interview guide for hospital social workers.

**Fig 2:**
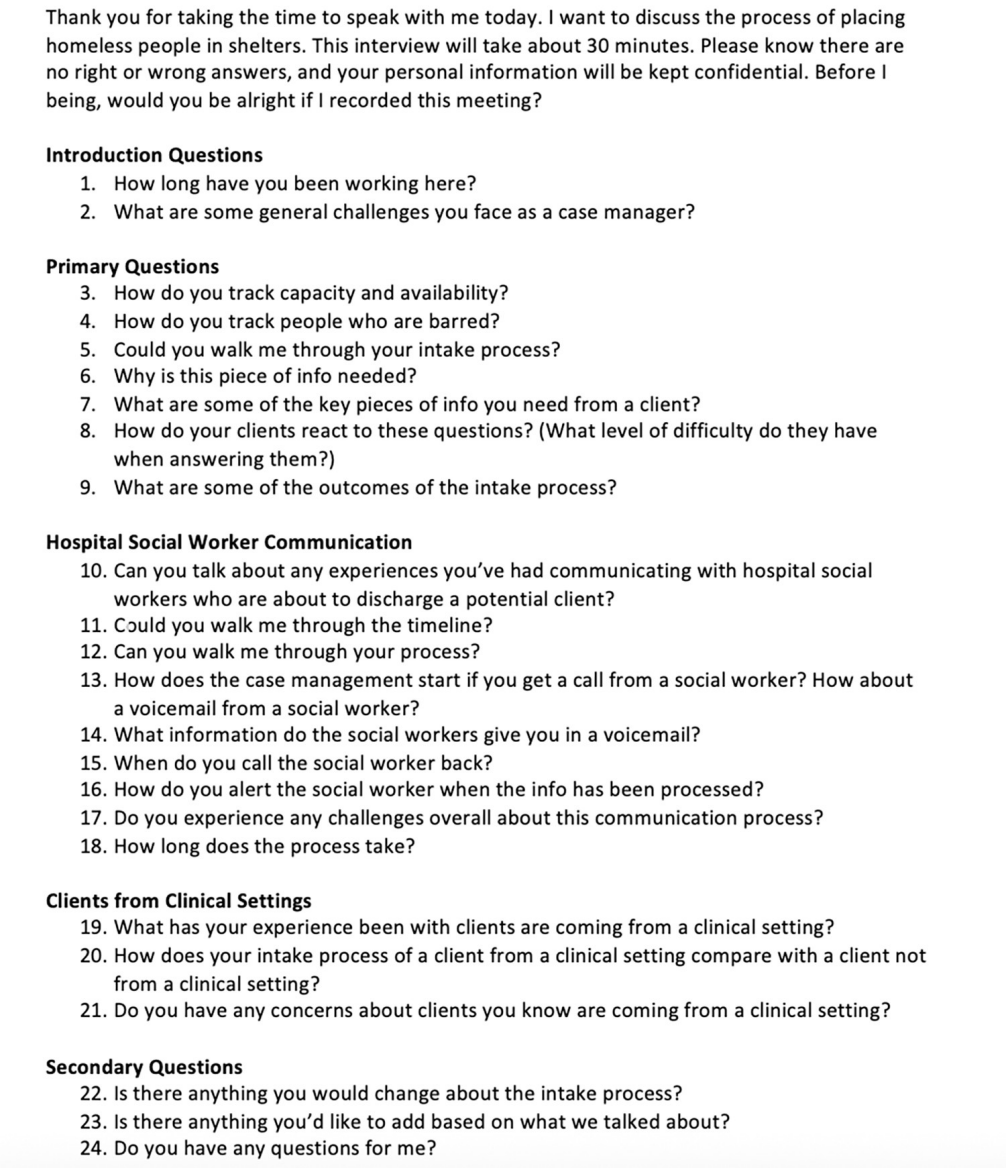
Interview guide for homeless shelter case managers.

Our team performed a qualitative design study to better understand the discharge and intake process for patients experiencing homelessness. We explored the discharge process at one large urban academic medical center and the intake process at four emergency shelters by interviewing ED social workers and shelter case managers. Ten subjects were interviewed, including three ED social workers and seven shelter case managers from various shelters in the Boston area. Interviews were conducted either through video chat or in person in the hospital for social workers or in the shelters for case managers. Two team members were involved in in-person interviews: one team member facilitated the interview and the other captured the interviewee’s responses. Interviews that were conducted through video chat were recorded and transcribed. Each interview lasted approximately 60 minutes. Interview guides for social workers and case manager were developed (Figs 1 and 2).

**Fig 3:**
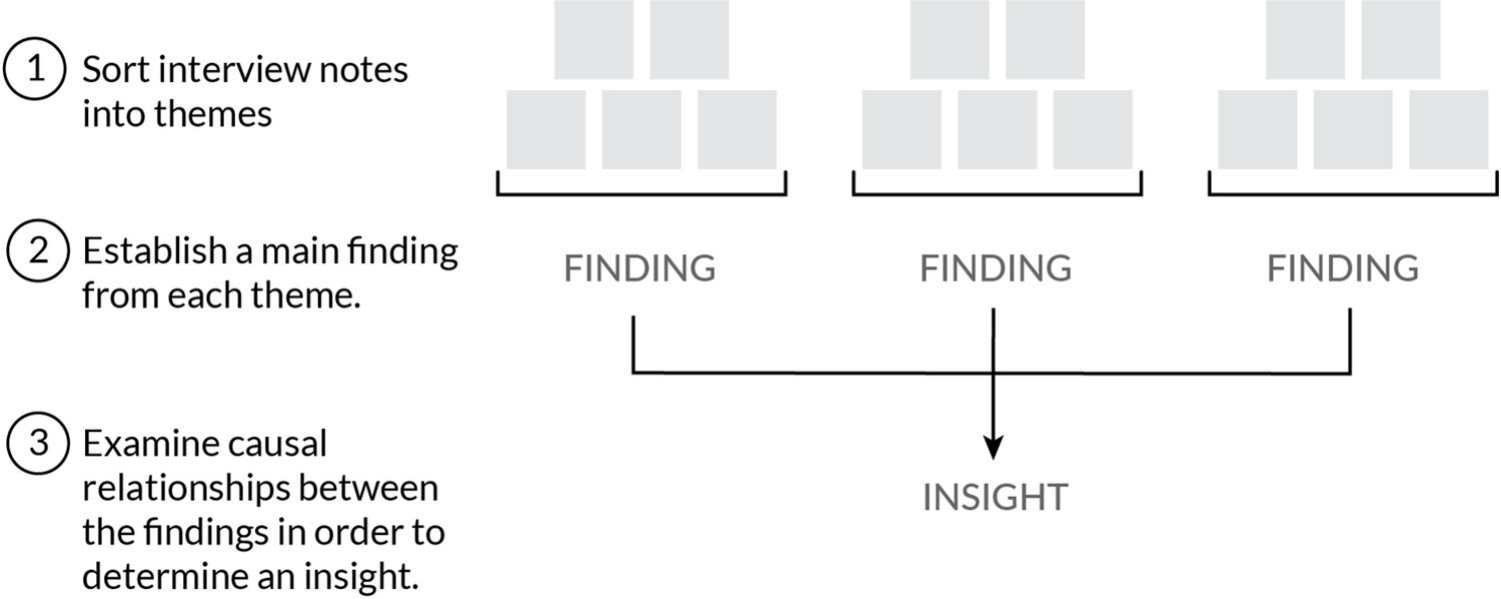
Illustrative diagram of summary analysis process.

The transcribed interview notes were analyzed using thematic qualitative analysis (Fig 3) as described by Braun and Clarke [15]. This method involves sorting transcribed phrases into themes. Themes were determined by grouping similar phrases, key words, or ideas [16]. For example, all references made to complying to specific state policies could be a theme. The summary of what that theme encapsulates is a finding. Interviews were no longer conducted after no new insights emerged that affected the thematic categorization [17].

Social workers referred to individuals as “patients,” while shelter case managers referred to them as “clients.” The social workers were interviewed to better understand their process of discharging a homeless patient. They were asked to describe the process of performing a discharge assessment as well as the specific questions they asked their patients. We sought to understand how their patients reacted to these questions and some challenges they experience when completing the discharge assessment. In addition, we wanted to know what the communication process with the shelter entailed and any challenges they faced during this process.

The shelter case managers were interviewed with the goal of understanding how shelter case managers handle a client recently discharged from the hospital. We also asked for an overview of the different types of shelter in the greater Boston area as well as an introduction to their intake process when they are assessing a potential new client, which we described in detail below. We asked about the interactions shelter case managers had with ED social workers, and their experiences with intaking clients who have been recently discharged from the ED.

This study was reviewed and approved by our hospital’s Institutional Review Board (Protocol Number: 2021P003576).

## Results

We learned about the critical disambiguation that must be made between emergency and non-emergency shelters. Non-emergency shelters are long-term facilities that provide a wide variety of services, ranging from day programs to washing machines. Accessing a non-emergency shelter requires an extended application, and often takes weeks before an individual is accepted into the system. This process is not one that homeless individuals are allowed to initiate on their own and requires the assistance of a social worker or case manager.

In contrast, an emergency shelter is one that will take individuals directly from any entry point–often the street–and does not require an extended application. They often serve as a bridge to a non-emergency shelter and require individuals to be able to perform activities of daily living (ADLs). Most emergency shelters have a limit on how long a PEH, i.e. the “client,” is permitted to stay. This paper focuses on discharge of PEH to emergency shelters specifically.

**Fig 4:**
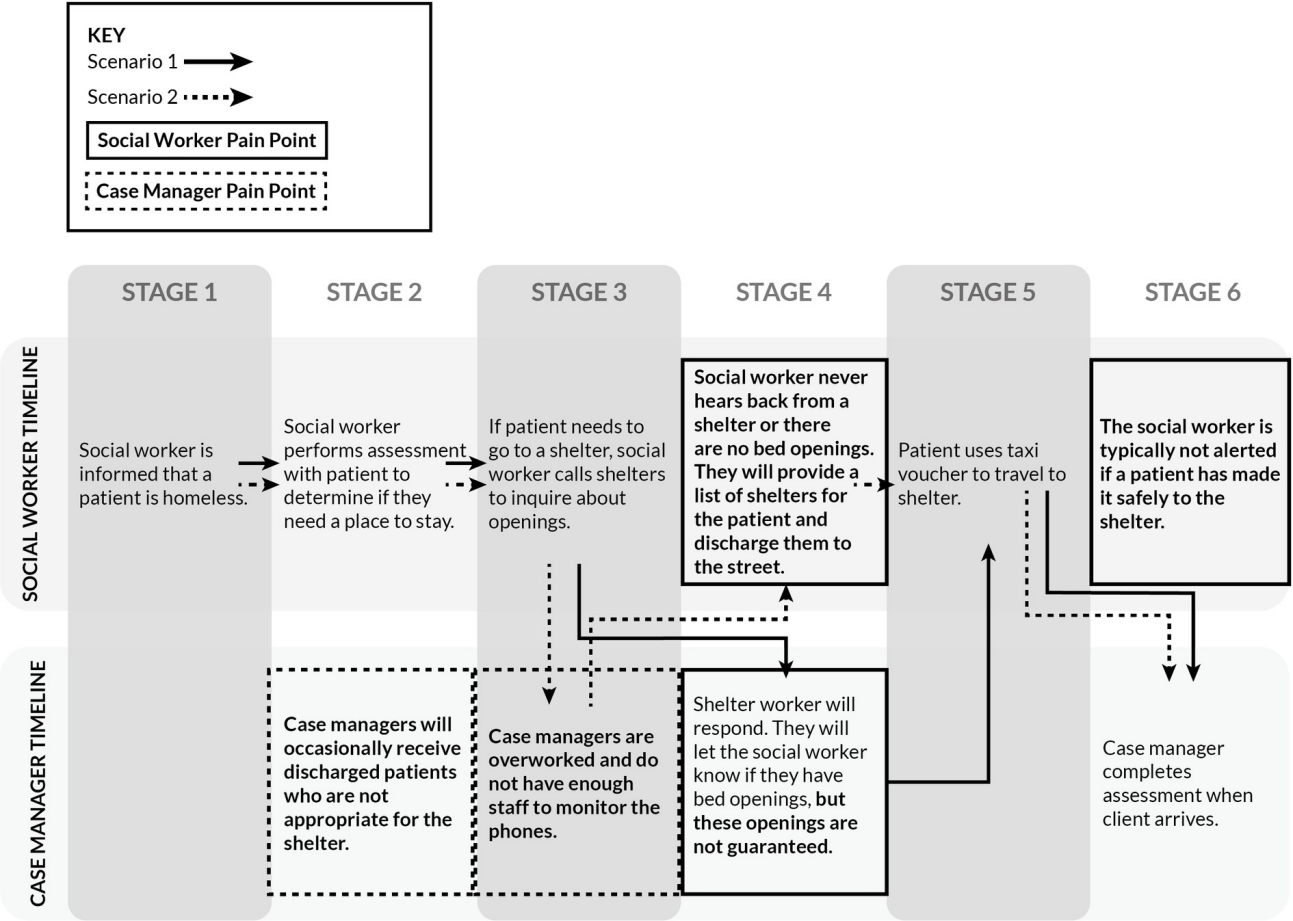
Timeline of the social worker’s assessment process aligned with the shelter case manager intake process.

Two processes arose in the data, the first being the discharge assessment process that the ED social workers perform, and the second being the intake assessment process that the shelter case managers perform (Fig 4).

### Discharge process for social workers

When a healthcare provider learns that a patient in the ED is homeless and is likely to be discharged, the provider will consult a social worker to determine an appropriate place to direct their patient, with the hopes of avoiding discharging any individual to the street. This process sometimes occurs simultaneously with the patient’s clinical evaluation, diagnostic testing, and treatment.

We describe in detail the steps of the evaluation process done by the ED social workers below.

### Patient interview

The social worker will begin by asking where the patient has been staying and if they are able to stay with family and friends. They will also take note of their age and family status, as there are some shelters that specifically accept only youths and young adults aged 24 or younger, or clients with families. It is also important for social workers to note if the patient has any substance use disorders, as some shelters do not allow clients with substance use disorders to be placed in their shelter. Importantly, they will inquire if the patient has been barred from any shelters. This assessment process typically takes between 20 and 30 minutes.

### Calling shelters

The social worker will then call emergency shelters to inquire about open beds. They typically do not get an immediate response. In general, social workers receive a response after half a day, but sometimes it can take up to two days. In their experience, larger emergency shelters are more responsive than smaller shelters.

### Intake process

If the social worker is able to reach a shelter case manager, the social worker will communicate the information they gathered from their assessment to help the shelter manager begin their intake process. Alternatively, the shelter case manager may ask to speak with the patient directly or may ask the patient to complete the intake once they arrive in person at the shelter.

### Discharge from the emergency department

Once the patient is ready for discharge, the social worker will give the patient a cab voucher to travel to the shelter. It is not typical for social workers to be alerted if their patient has safely made it to a shelter. If the social worker is unable to find an available bed for the patient, the provider will discharge the patient to the street with a list of shelters.

### Intake process for case managers

Once a client arrives at the shelter, the case manager will perform an intake assessment to determine if the client is an appropriate fit for the shelter. This decision is based on a variety of factors, including medical or psychiatric issues that would prevent a patient from caring for themselves. Case managers expressed a desire for hospital social workers to conduct a thorough assessment of their patient in order to avoid patients with complex medical needs being inappropriately sent to a shelter. In cases when the fit is deemed inappropriate, the case managers will send the client to a medical facility that can better address their needs.

One of the goals of the intake process is diversion. This means that case managers attempt to, in collaboration with the client, find a safe alternative to staying at an emergency homeless shelter. One case manager mentioned that they “can usually find the client a place to stay with a family or friend if they are newly homeless.”

To facilitate diversion, case managers will specifically craft their questions to better understand their client’s background and the resources available to them. For example, instead of asking, “Can you stay with another friend or family member?” which warrants a yes or no answer, they will ask “Who are some friends you’ve spoken with in the past few months?”. They counsel clients on assistance programs that are available; one such example is a stipend for groceries offered to a potential host in order to ease any tensions that may arise from housing a friend or family member.

One challenge case managers faced during the COVID-19 pandemic was that it forced shelters to decrease their capacity. With limited capacity, many shelters began to accept patients on a first-come, first-served basis. There have been cases when hospital social workers communicated that they would be sending a patient, but the patient never arrived, resulting in a shelter bed being held for them inappropriately. As a result, some shelters decided to prioritize giving available beds to clients who were already physically present at the shelter, rather than holding beds based on phone calls, some of which may be from hospital social workers.

### Insights

There is a substantial amount of information required for the shelter intake process. As such, ED social workers often do not have the bandwidth or time to collect the information that a case manager would need. As one social worker noted, “talking to patients to do comprehensive assessment takes 20–30 minutes.” Furthermore, the process of securing a bed for a patient is lengthy, often resulting in the ED being ready to discharge a homeless patient before social workers are able to secure housing, which increases the chance of a patient being discharged to the street.

Next, due to the new first-come, first-served protocols adopted by emergency shelters as a response to COVID-19, having a social worker call ahead to confirm bed availability may be a potential waste of time if the homeless patient does not arrive in a timely fashion. One social worker commented that “placement for shelters is hard because it’s not like detox placement. We can call shelters, but we don’t know 1. if they’re barred, 2. if they get in, if they even go there, because it’s first-come-first served.” Another social worker noted that some emergency shelters don’t answer their phones, so “patients have to be sent without a guarantee of acceptance.”

**Fig 5:**
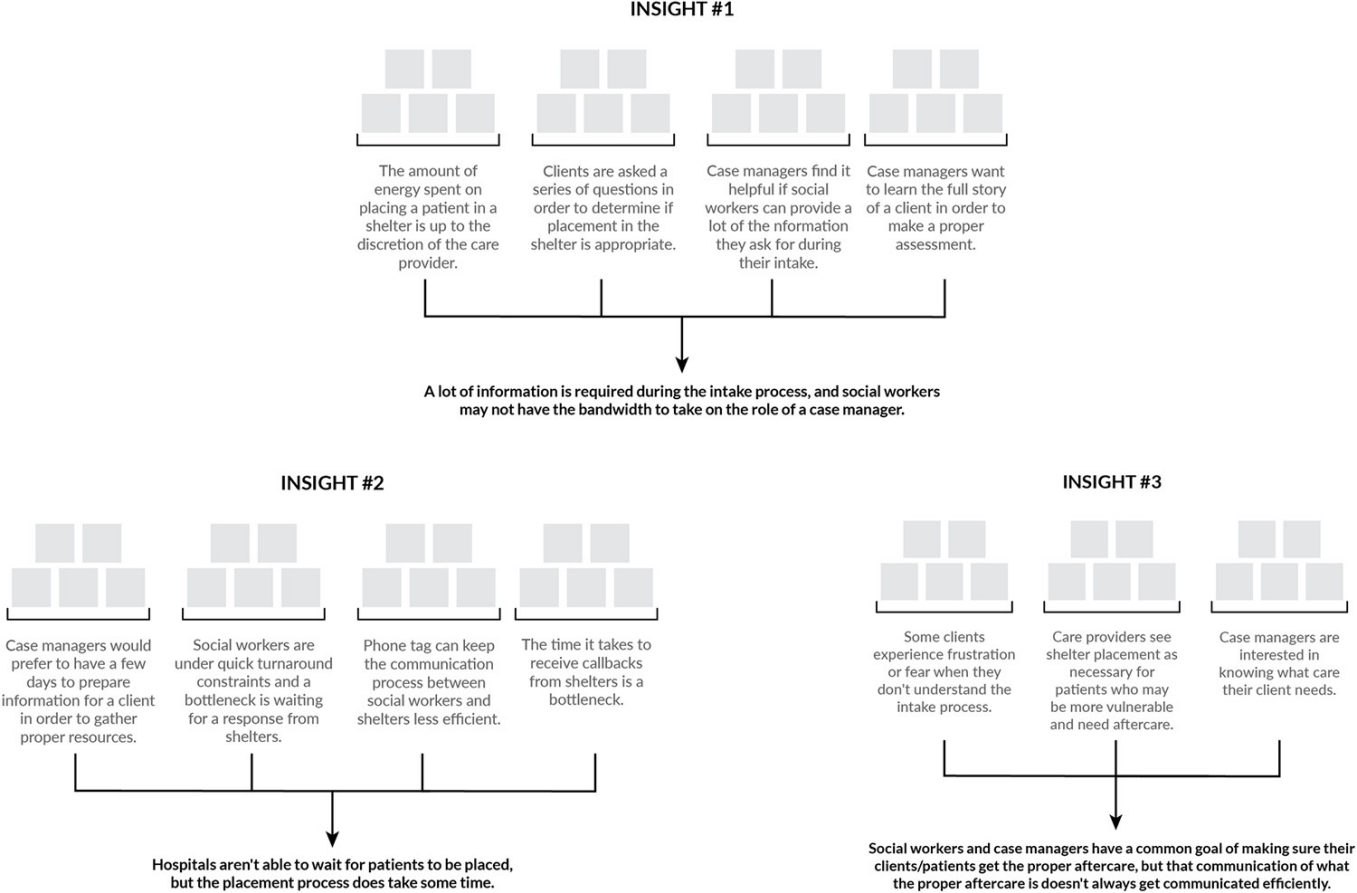
Summary of key challenges identified in our study.

Finally, social workers and case managers share the common goal of ensuring that their patients or clients can recuperate from their ED visit with proper aftercare, but the understanding of what constitutes proper aftercare doesn’t necessarily get communicated efficiently. While social workers reported asking about patients’ ability to perform their ADLs and documenting medical needs such as oxygen and wheelchair access, case managers reported that they often receive clients from the hospital who have higher medical and accessibility needs that the shelter cannot meet. We summarize the primary challenges identified in our study in Fig 5.

### Social worker findings

Social workers in the ED are the first point of contact when it comes to assisting PEH with housing difficulties. Based on the interviews conducted, we uncovered some of the challenges that ED social workers must overcome to aid their patients. Of note, social workers revealed that the amount of energy spent on placing a patient in a shelter is up to the discretion of the care provider and can vary greatly depending on the patient load at any given time. There is no state mandated protocol for the discharge process (other than the right to shelter for families and pregnant people [18]) and therefore no state-wide standardization among ED social workers or even healthcare facilities. This results in patients receiving varying care, depending on time of day, ED overcrowding, and other factors outside their control.

Another challenge brought to light is that social workers are under quick turnaround constraints, since there is a limit to the amount of time that a patient can spend in the ED before they are discharged. Although the patient interview often happens simultaneously with the medical workup, efforts to improve ED throughput sometimes results in homeless patients being ready for discharge before a shelter has been arranged for them by social workers. Heavy case load, extensive interviews and late identification of a patient as a PEH make the time constraint challenging. This is further exacerbated by the fact that a callback from a shelter can sometimes take “up to two days”, as our interviews revealed; this was specifically highlighted as it poses a significant bottleneck in the social worker discharge process. Furthermore, they noted that a lack of staff manning telephones makes the communication process between social workers and shelters less efficient. A social worker commented that “sometimes [they] just can’t even get in touch with shelter staff.” Another social work noted that “it would be helpful to have a running list of beds that are open and at what distance they are.”

Lastly, social workers expressed frustration that there is no closed loop communication regarding the outcome of a discharge they helped facilitate, since they are unable to find out if a patient made it to a shelter and if they were appropriate for it. One social worker commented that they only receive confirmation that a patient arrived safely at a shelter in “1 out of 50 cases.”

### Shelter case manager findings

In terms of triaging PEH for shelter placement, the interviews with both groups revealed that shelter case managers and social workers see placement as necessary for patients who may be more vulnerable and need aftercare. Based on our discussions, “vulnerable” is not defined as a patient who cannot care for themselves, since most individuals are already deemed as capable of completing their ADLs. Rather, it is a patient that has experienced a medical problem that, if not cared for, will likely cause them to revisit the ED.

Based on our interviews, shelter managers would find it helpful if social workers could provide a lot of the information they ask for their intake. This would speed up the intake process once the patient arrives at the shelter. For instance, a main initial goal of the case manager’s assessment is to determine if placement in the shelter is the most appropriate; they do so by evaluating each individual situation to see if diversion to an alternative housing situation, such as staying with a friend or family member, is possible. One case manager mentioned that “clients who are newly homeless most likely have a different place to stay that they didn’t originally think of.” Case managers reported that it would be helpful if social workers could ask some of these diversion-based questions.

In addition, case managers endorsed that communication regarding any further information, such as follow-up care needs, would be beneficial as well. These focused questions would help ensure that patients who are being sent to a specific homeless shelter are appropriate for that facility, thus saving case managers from unnecessary intake processes. One shelter case manager commented:

“I feel like [hospital] dischargers get desperate and hear the shelter saying ‘maybe’ or ‘not a hard no’ and just discharge the person. The level of care that we can provide is ‘emergency shelter’–we’ve had to discharge someone from our shelter because they’re not going to be able to get in and out of bed. They can’t toilet on their own. They have an oxygen tank. They have long term illnesses. What we are then faced with if they get dropped off with us is that we’re either saying past 4:30pm on a Friday because we’re diverting them back to a hospital, or they are going outside. That’s the nightmare scenario we face all the time.”

To avoid such scenarios, case managers shared that they would prefer to have a few days to prepare information for a client to gather proper resources. This would be useful in cases where the client may have a substance use disorder and need additional support.

Finally, shelter managers reported that some clients experience frustration or fear during the intake process. This can be for a variety of reasons, including the volume of questions and possible confusion over the fact that an assessment is needed at all. One case manager noted: “People get frustrated over the phone like teenagers who don’t want to answer questions or don’t realize that they do have other options and just want to say they’re homeless. I start by asking them ‘So what’s been going on? Where did you sleep last night?’ And then we work our way back.”

### Data dictionary

Based on our discussion with both social workers and case managers, we determined some of the criteria that are used to assess whether a PEH is an appropriate fit at a shelter. For simplicity, the PEH is referred to as the patient in this context. We have assembled this information into a data dictionary, which is presented in Table 1.

**Table 1:** Data dictionary compiling the most common criteria used by emergency shelters to assess potential patients.

Field Name	Data Type	Description	Response Options
Gender	List	Gender of patient	male/female
Accompanied by family	Boolean	Accompanying family is present?	yes/no
Returning patient	Boolean	Is the shelter only accepting patients who have stayed with them in the past 2 weeks?	yes/no
Age	Number	Age of patient	integer
Substance use disorder	Boolean	Does the patient have a substance use disorder?	yes/no
Barred	List	Is the patient barred from a particular shelter?	yes/no/unaware
Bunk availability	Boolean	Is there a bunk open at the shelter?	yes/no
Bunk type	List	Does patient require a top or bottom bunk?	top/bottom/either
Veteran status	Boolean	Is the patient a veteran?	yes/no
Language	List	What is the patient’s primary language?	English, Creole, Cape Verdean, Spanish
Ability to care for themself	Boolean	Is the patient able to care for themself?	yes/no
Resident restrictions	Boolean	Was the patient’s last place of residence within the same city as the shelter?	yes/no

#### Gender

Many shelters do not house those identifying as male and female in the same shelter.

#### Accompanied by family

Massachusetts is a right to safe shelter state for families [18]. This information is important for social workers because it enables them to draw on additional resources, such as rehousing and assistance with rental payments.

#### Returning patient

During the COVID-19 pandemic, a few shelters had a policy to only accept patients who had stayed with them in the past 2 weeks.

#### Age

There are some shelters that are only available for minors. Shelters have varying age cutoffs for being considered a minor, which range between 18 and 25 years of age.

#### Substance use disorder

Some shelters will not accept a patient with a substance use disorder.

#### Barred

Each shelter keeps their own list of who they will not allow back at their shelter. An action that results in barring usually involves a violent act.

#### Bunk availability

The ability for a shelter to take on a new patient is dependent on how many bed spaces the shelter has available.

#### Bunk type

If the patient isn’t able to access a top bunk, there must be a bottom bunk available.

#### Veteran status

Some emergency shelters will only accept a patient if they are a veteran.

#### Language

Some patients may not speak fluent English and require a shelter where a staff member can provide assistance in their preferred language.

#### Ability to care for themselves

Emergency shelters require that all patients are able to perform their ADLs.

#### Resident requirement

Some Boston emergency shelters require that the patient’s most recent place of residence be in Boston proper.

## Discussion

The value of this research comes from the knowledge that ED interventions have been shown to play a significant potential role in breaking the cycle of homelessness [19]. In keeping with prior research, our interviews suggest that the hospital discharge process, which should work to streamline a patient’s safe return to the community, has been found to be especially difficult and ineffective for PEH [20]. Current research focusing on the discharge process of PEH is scant, and recent papers by Canham and colleagues as well as Jenkinson and colleagues call for further elucidation of the discharge process [8, 21], which our research aims to do. In particular, Jenkinson and colleagues point out that the entire discharge to intake process is not well described in literature [8]. The specific, actionable information provided by these interviews could be advantageous in further improving discharge planning at both ends.

Our findings underscore the need for better coordination between hospitals and external institutions. One of the main themes that emerged from our interviews was the lack of clear communication between hospital social workers and shelter case managers, with the outcome being patients being inappropriately discharged to a shelter that cannot meet their needs. It was interesting to hear from case managers that they would want few days to properly evaluate whether a client would be appropriate for their facility. While this is an important consideration and can be achievable when a patient is being discharged from an inpatient setting, ED discharges are generally same day, making this difficult. Recognizing the constraints of discharge planning is only the first step. Current literature discusses not only the utility of specific training on discharge planning, but also of understanding the issue in the context of different sectors (i.e. healthcare settings and social resources, including shelters) and achieving better integration between them [22].

Our institution has the opportunity to improve continuity of care by developing stronger collaborative relationships with outpatient clinics and municipal agencies. A Chicago-based study by Sadowski and colleagues randomized PEH with chronic medical illnesses in a program to an intervention that provided longitudinal case management services from both hospital and transitional housing-based social workers versus usual care. They found that patients in the intervention group had a reduction in both ED visits and hospitalizations, and were more likely to be placed in stable housing. It is worth noting that intervention social workers had caseloads of 20 or less, which might not necessarily be replicable in all settings [23]. Another study out of Australia by Wood and colleagues found that a collaboration between Perth’s Housing First Programme, a specialist homeless primary care practice, and a hospital-based clinical team reduced the number of ED visits and hospitalizations for their intervention group, and led to cost savings for the health system [24].

Shelter case managers noted that patients are often overwhelmed by all the questions being asked from different parties. A streamlined intake process may be beneficial in alleviating some of the perceived distress. This observation by shelter case managers also raises the important point that insight from PEH would be monumental in furthering our efforts; we explore this topic briefly in the Limitations section below.

### Limitations and implications

Although this paper fills a gap in current literature by examining the discharge process from emergency department to homeless shelter in detail, it does have limitations. Our small sample size of three ED social workers and seven shelter case managers, as well as the regional variability in municipal and state resources available to PEH, limit the generalizability of our study. However, other qualitative studies exploring hospital discharge processes for this population have yielded similar insights. A thematic analysis by McCormack and colleagues found that in resource-deprived settings in England, hospital-based staff felt time constraints and the pressure to “free up beds” translated into poor or incomplete discharge planning for PEH. Participants also perceived that outpatient medical care is often unable to meet the complex needs of PEH [25]. Our findings suggest that these barriers are not limited to resource-deprived settings and can affect PEH in large urban and relatively well-resourced settings, where the majority of PEH reside [26].

Another limitation is that our study did not involve interviews with PEH, which provide a crucial perspective into the barriers in care coordination after ED discharge. In a qualitative study out of Australia by Strange and colleagues, thematic analysis identified key factors influencing the decision by PEH to seek outpatient medical care, which included doctor-patient empathy and an understanding of their unique needs by the medical teams [27]. A study by Ramsay and colleagues interviewed PEH in Niagara, Canada, and found that barriers to outpatient care included systemic factors such as cost of care, transportation, and accessibility, as well as patient-provider factors such as lack of trust and poor therapeutic relationships [28]. In order to succeed, ED and hospital discharge planning interventions need to account for the preferences and unique circumstances of PEH.

### Potential role of technology

While establishing collaborative relationships between hospitals and community resources is paramount to ensuring continuity of care, it is also important to consider how technology can enhance those partnership and improve their sustainability. For instance, to address one of the challenges raised by ED social workers (time constraints due to speedy discharge), ideally a patient would be flagged as homeless immediately upon presenting in the ED, giving both the emergency physician and the social worker ample time to organize disposition in the event that the patient is to be discharged. This could be accomplished by utilizing the capabilities of electronic health records, such as establishing automatic “flags” via natural language processing for patients who have had a history of homelessness documented in their previous encounters, or by using address validation software, an approach that has been validated in the processing of Social Security Administration claims in the United States [29].

A standardized data dictionary, as defined in our research, is pivotal for several reasons in the evolving technological landscape. Firstly, it facilitates uniformity in data representation across diverse platforms, such as EMR integrations, mobile applications, and government technological solutions. This standardization is crucial for ensuring that various systems ‘speak the same language,’ thereby enhancing interoperability. Secondly, a well-established data dictionary can catalyze technological outcomes by providing a clear framework for developers and policymakers, guiding the development of more effective, user-friendly tools for both providers and PEH. For instance, with standardized data definitions, mobile applications can be more effectively tailored to assist PEH in locating resources or receiving healthcare follow-up reminders. Similarly, EMR systems could be better integrated with shelter bed management software, reducing the ‘phone tag’ and significant bottlenecks currently experienced, as our research has identified. A consistent data framework could also assist in developing government technological solutions that are more adept at addressing the real-time needs of PEH [30].

Technology could be further leveraged to facilitate communication between ED social workers and shelter case managers. A potential solution could be a digital platform that allows shelters to keep track of their bed availability in real time so that ED social workers know which shelters to call. However, this would require significant investments in technical equipment, staffing, and training, as shelter staff would be in charge of keeping the platform updated in real time in order for this solution to work. Furthermore, it can be challenging to engineer crosstalk between different systems such as hospital electronic health records and shelter bed management software.

There might also be a role for algorithmic matching of patients experiencing homelessness to shelter beds. Algorithms have been successfully used in the allocation of scarce resources in healthcare settings, such as in the distribution of personal protective equipment to healthcare facilities during the early COVID pandemic [31]. A digital platform that algorithmically matches patients experiencing homelessness to appropriate shelters that are most likely to have availability on a certain day and at a certain time could decrease the time and effort on behalf of ED social workers as well as the patient’s length of stay in the hospital. Research has shown that PEH are at high risk of returning to the emergency department [21, 32]. Therefore, efforts to prioritize the placement of patients based on “vulnerability”—a concept raised by both our social workers and case managers during our interviews—are important to highlight. An algorithm that can triage patients based on risk of re-presenting to the hospital could be useful for both social workers and case managers.

A digital solution that enables shelter case managers to give feedback to ED social workers whenever a patient is inappropriately sent to their shelter could lead to improved satisfaction on both ends. Currently, states and communities employ Homeless Management Information Systems (HMIS) that collect client-level data on the provision of housing and services to homeless individuals. The Department of Housing and Urban Development requires homeless service systems (known as Continuums of Care) to use an HMIS for government reporting and system monitoring [33]. These already existing tools could be harnessed to provide a means of communication between ED social workers and shelter staff in addition to phone calls, which might accelerate communication between both parties.

Lastly, if the discharged patient has access to a mobile device, the information from the digital solutions mentioned above could potentially be used to inform the discharged patient on resources for PEH, shelter bed availability, and healthcare follow-up reminders. A 2017 study of PEH in Los Angeles and Long Beach, California found that the majority of respondents owned a cell phone currently (94%) or in the past 3 months (97%) [34].

## Conclusion

People experiencing homelessness face many challenges finding shelter after an emergency department visit. ED social workers and shelter case managers work together to find appropriate placement for PEH, but the current process for discharging patients experiencing homelessness from the ED has several bottlenecks. As our extensive discussions with ED social workers and shelter case managers revealed, ED social workers have limited time and bandwidth to collect all the necessary information that shelter case managers need. They are also under pressure to procure placement by the time a patient is ready for discharge from the ED in order to facilitate patient throughput.

Both ED social workers and shelter case managers agree that communication throughout this process is fragmented and inefficient, leading to patients occasionally being sent to a shelter that cannot meet their needs. By better understanding the current discharge process of PEH from the ED and identifying its bottlenecks, we hope to open the door to innovative solutions that can improve care for these patients during and after their ED visit.

## Abbreviations

PEH: people experiencing homelessness
ED: emergency department
ADLs: activities of daily living
HMIS: homeless management information system
